# Mediastinal teratoma with hydrops fetalis in a newborn and development of chronic respiratory insufficiency

**DOI:** 10.2478/raon-2013-0080

**Published:** 2014-11-05

**Authors:** Milanka Simoncic, Silvo Kopriva, Ziva Zupancic, Maja Jerse, Janez Babnik, Matevz Srpcic, Stefan Grosek

**Affiliations:** 1 Department of Pediatric Surgery and Intensive Care, University Medical Centre Ljubljana, Ljubljana, Slovenia; 2 Institute of Radiology, University Medical Centre Ljubljana, Ljubljana, Slovenia; 3 Institute of Pathology, Faculty of Medicine, Ljubljana, Slovenia; 4 Department of Perinatology, Unit for Neonatal Intensive Care and Therapy, University Medical Centre Ljubljana, Ljubljana, Slovenia; 5 Department of Thoracic Surgery, University Medical Centre Ljubljana, Ljubljana Slovenia

**Keywords:** mediastinal teratoma, non-immune hydrops fetalis, diaphragm paralysis, chronic respiratory insufficiency

## Abstract

**Background:**

Mediastinal fetal teratoma can be detected as a mass in the chest during a routine prenatal ultra-sound screening. Because of the pressure on mediastinal structures it can be the cause of non-immune hydrops fetalis and polyhydramnion. The development of hydrops fetalis leads to fetal death or premature delivery in most reported cases. Early surgical removal is important, but, the result of treatment depends on the stage of development of mediastinal organs and complications in the postoperative period.

**Case report.:**

A 31-year-old *gravida* carrying twins, with spontaneous membrane rupture at 32 weeks gestation underwent urgent caesarean section after antenatal ultrasound revealed severe polyhydramnion and hydrops fetalis in geminus A. The child was intubated immediately after birth due to severe respiratory distress. Ultrasound and X-ray revealed a tumour mass in the right hemithorax. Tumour resection was performed at the age of 7 days. Histology examination revealed an encapsulated immature teratoma. The postoperative course was complicated with respiratory insufficiency which turned into chronic at the age of eight months.

**Conclusion:**

This is the fifth reported child with fetal mediastinal teratoma and severe hydrops fetalis that survived the neonatal period. Additional diagnostic search revealed abnormal course of both pulmonary arteries, which was probably one of the main causes of respiratory insufficiency.

## Introduction

Mediastinal teratomas are the second most common extragonadal teratomas in children.^1^ There are some reports of mediastinal teratomas discovered as a mass in the chest with antenatal ultra-sound (US) in fetal life.^2^–^8^ Rapid growth in fetal life can compress lungs, heart and great vessels, which can lead to development of non-immune hydrops fetalis (NIHF).^7^ High mortality can be prevented only by an early intervention at the onset of NIHF that dramatically improves deteriorating condition and survival rate of fetuses.^2^–^4^

If fetus with mediastinal teratoma survives until birth, tumour compression on mediastinal structures usually manifests with severe respiratory distress in newborn.^19^–13 Early surgical excision is important, but outcome depends on several additional factors such as lung hypoplasia, the stage of heart development, tracheomalacia and peri- and postoperative complications.^3^,^6^–^8^,^10^ We present a case of mediastinal teratoma in a newborn associated with severe NIHF with several complications after birth and surgical procedure which led to the development of chronic respiratory insufficiency. This is the fifth reported case of a mediastinal teratoma with NIHF in which the newborn survived the neonatal period.

## Case report

**FIGURE 1.**
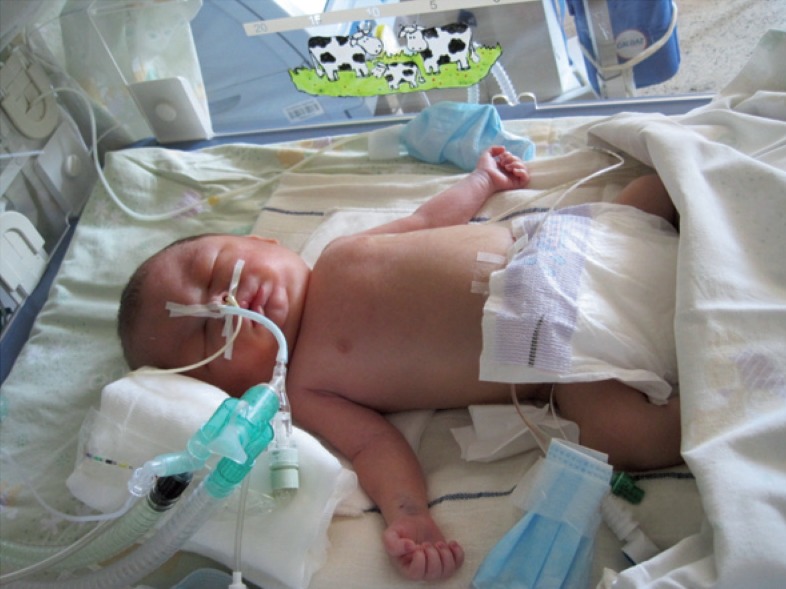
Geminus A with severe hydrops fetalis was intubated immediately after birth.

A 31-year-old *gravida* 2 with twins, *para* 1, was referred to the University Medical Centre Ljubljana because of spontaneous membrane rupture at 32 weeks gestation after an uncomplicated pregnancy. After admission US revealed polyhydramnion and severe NIHF in geminus A. Due to life threatening condition also for healthy geminus B an urgent caesarean section was performed. Geminus A was a boy with Apgar score 3/5 and a birth weight of 3,200 g. He was intubated immediately after birth because of severe NIHF and respiratory distress (Figure 1).

**FIGURE 2.**
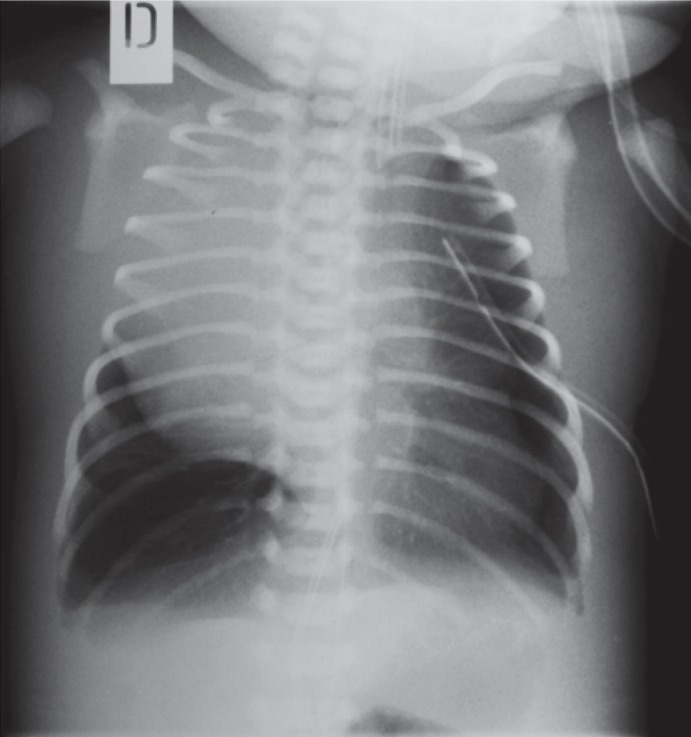
Chest x-ray after birth demonstrates large round tumour in the right hemithorax. (6 × 7 cm) with deviation of mediastinal structures to the left. Soft tissues of the thoracic wall are oedematous.

An initial arterial blood gas analysis revealed a respiratory acidosis with a pH of 6.91, PaCO_2_ 19 kPa and HCO_3_ of 13 mmol/l. US revealed anterior mediastinal cystic mass extending into the right and left hemithorax with marked pleural effusion bilaterally. Chest X-ray confirmed a homogenous mass in the anterior right hemithorax with shifting of the mediastinum to the left (Figure 2). Ductus arteriosus with left to right shunt was still patent. Because of pleural effusion pleural puncture was performed, further complicated by pneumothorax. Despite ventilation with high frequent oscillations respiratory acidosis still persisted with PCO2 between 10 and 15 kPa. Due to low blood pressure (mean arterial pressure 4 kPa) and very poor urine output he needed vasoactive support with dopamin and dobutamine. US of the heart showed signs of pulmonary artery hypertension. The diagnosis based on radiological finding was congenital cystic adenomatoid malformation, which needed surgical excision on day seven of life.

Right anterolateral thoracotomy was performed with removal of soft policystic encapsulated tumour without invading into the surrounding structures. Tumour was not connected to respiratory system and lungs were macroscopically normally developed. The surgical specimen measured 55 × 50 mm, weighed 54 g, and was extensively sampled.

**FIGURE 3.**
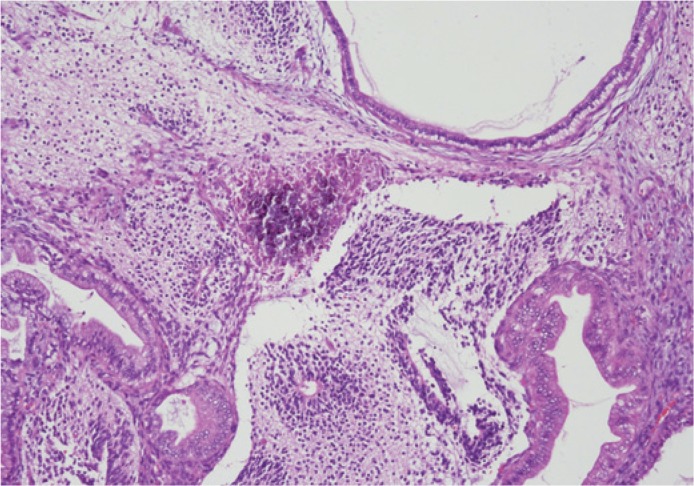
Focal calcification and the presence of cystic structures alternating with solid areas composed of mature and immature neural tissue.

**FIGURE 4.**
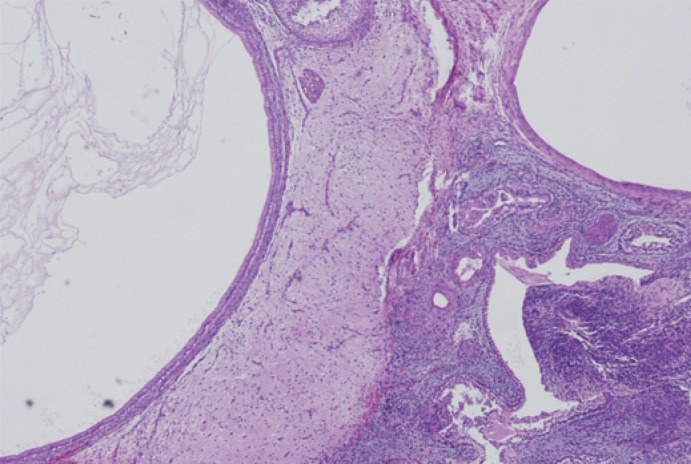
Cystic walls lined by columnar and cuboidal epithelium. Solid parts displaying brain tissue comprising of glia cells and primitive neuroepithelium.

**FIGURE 5.**
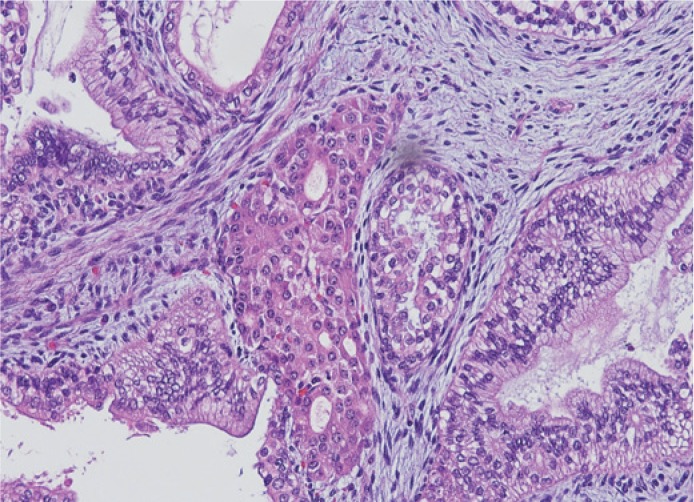
Gland-like structure with acinar epithelium resembling pancreatic tissue. Cystic formation embedded in a loose immature stromal tissue.

Microscopically the tumour was predominantly, in 70%, composed by immature tissue derived from different germinal layers. Cystic areas were alternating with solid tissue. The majority of the tumour was characterized by immature neuroectodermal tissue. The elements of mature glial tissue, ganglion cells and structures of choroid plexus were also randomly interspersed. Mesodermal tissue was represented by mature muscle, bone, adipose tissue, and mature and immature cartilage components with sparsely cellular mesenchymal tissue. The mature components of the tumour were also represented by the microfoci of hepatic differentiation, and pancreatic tissue, as well as sparse elements in the form of skin appendages (Figures 3,4,5). Serum human chorionic gonadotropin and alpha protein were within normal limits, and no further oncologic treatment was necessary.

**FIGURE 6.**
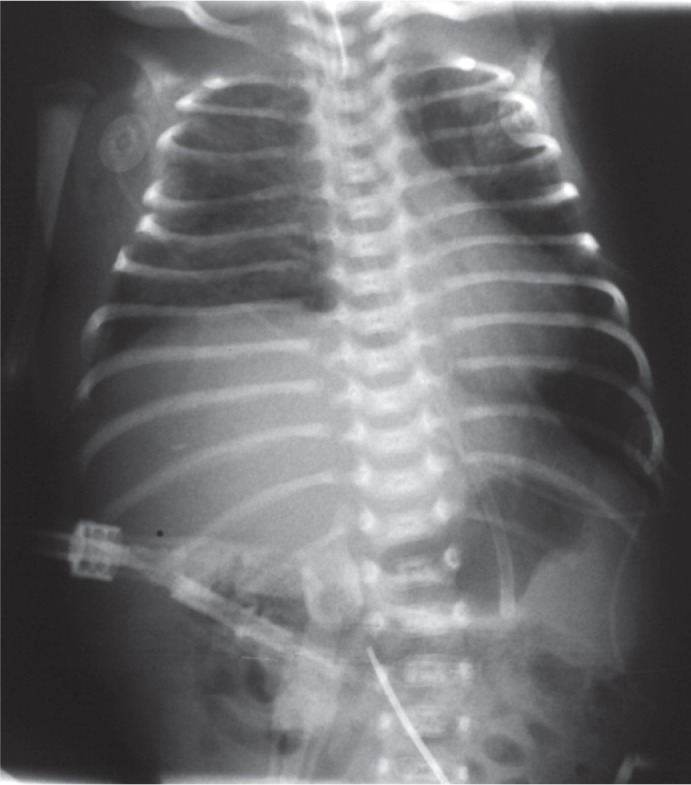
Chest x-ray demonstrates high position of the right diaphragm due to postoperative paralysis.

Hydropic oedemas subsided three days after operation. Urine output and hemodynamic parameters were stable and we discontinued the vasoactive drug support one week after operation. Postoperative course was complicated by a spontaneous pneumothorax on the right side and a sepsis caused by *Staphylococcus epidermidis*. Fifteen days after operation, the newborn still needed support with synchronized intermittent mandatory ventilation. He had multiple spontaneous episodes of oxygen desaturation. We performed diagnostic rigid bronchoscopy with which we excluded changes in the bronchial system. US of the heart showed signs of pulmonary artery hypertension, persistent ductus arteriosus and patent *foramen ovale*. We started treatment with nitric oxide and selective inhibitor of phosphodiesterase type 5, with no improvement. Chest X-ray revealed hyperinflation of the left lung and high position of the diaphragm on the right side (Figure 6).

With electromyography we confirmed phrenic nerve palsy and right diaphragm paralysis. At 50 days of age, a second surgery was performed and the right diaphragm was plicated. He was extubated successfully 1 week after plication. After extubation the cyanosis and tachipnoea were periodically observed with paradoxical movement of the chest. Scintigraphy of the lungs revealed smaller right lung without regional perfusion or ventilation defects.

At 3 months of age, he was referred to the intensive care unit with respiratory distress, intubated and mechanically ventilated. Chest X-ray revealed pneumonia and right lung atelectasis. Computed tomography (CT) scan of the lungs again revealed a high position of the right diaphragm and a consolidation of the right and left lung. Due to multiple failures of weaning from mechanical ventilation and extubation one month after admission the third operation was performed with right diaphragm plication and lung biopsy. Biopsy of the right lower lung lobe revealed acute interstitial pneumonia and alveolitis. After plication and antibiotic treatment, there was clear but slow improvement. He was extubated one month after plication and was well for one month.

At 6 months of age, he was again referred to the intensive care unit with dyspnoea, tachipnoea, cyanosis and severe respiratory acidosis with PaCO_2_ 10 kPa. Bronchoscopy revealed narrowing of the left inferior bronchus. US of the heart showed structurally normal heart with the appropriate function of both ventricles. For further assessment of the cardiovascular function heart catheterization was performed, revealing an abnormal course of both pulmonary arteries with signs of pulmonary artery hypertension. Right middle lung lobe was practically without normal right pulmonary vascularisation. With no improvement in respiratory condition one month after treatment we decided for a tracheostomy. He was breathing with nasal continuous positive airway pressure ventilation (CPAP) on Legendair ventilator (Covidien AG™) with the addition of oxygen FiO2 0.25–0.35) after the procedure. We disconnected him from the ventilator in order to evoke spontaneous breathing several times per day.

At 11 months of age, paediatric neurologist made a neurologic assessment. He had severe generalized muscle hypotonia, a convergent strabismus and normal proprioceptive reflexes. Babinski sign was still present. Magnetic resonance imaging (MRI) confirmed atrophic frontal brain changes. We excluded metabolic diseases.

At 1 year of age, the boy was discharged from hospital in a stable clinical respiratory condition with tracheostomy and Legendair ventilator. At the age of 2 years, bronchoscopy revealed massive granulation in trachea just below the tracheostoma. After removal of the granulation the tracheostoma was closed and he is breathing on his own.

## Discussion

Mediastinal tumours can be detected before birth by routine antenatal US, most frequently during second and third trimester. With an US we can determine the location of the tumour, pressure on mediastinal structures, cystic or solid tumour components, development of NIHF and polyhydramnion.^14^ With fetal MRI we can obtain more precise information about the tumour and its relationship to adjacent structures.^2^,^4^,^14^ Neonatal outcome depends on the size and location of the tumour, presence of NIHF and early prenatal or perinatal intervention.^12^–^4^,^13^

Liang *et al*. evaluated the influence of intrathoracic mass on fetal hemodynamics with Doppler flow velocimetry. They showed in their case that the main changes occurred in the heart and great vessels.^5^ On the other hand NIHF indicates that tumour causes intrauterine pressure on the heart and major blood vessels.^7^ It is expressed with oedema, ascites, pleural effusion, and hepatomegaly.^2^,^3^ NIHF and polyhydramnion are poor prognostic signs.^3^ Pressure in the thorax leads to the development of heart dysplasia and lung hypoplasia with reported death prenatally and after birth.^7^,^8^,^15^,^16^ Polyhydramnion develops due to pressure on the oesophagus and decreased swallowing of amniotic fluid.^17^

We found only four reported cases with prenatal identified NIHF caused by mediastinal teratoma, who survived the neonatal period (Table 1).^2^–^4^ In these cases different approaches were used for a successful outcome. In the case by Takayasu *et al*. NIHF resolved immediately after aspiration of the tumour cyst.^4^ Merchant *et al.* recommend *in utero* resection of tumour if NIHF develops before 30 weeks gestation. After 30 weeks gestation it is important to evaluate lung development and airway compromise. If the child’s airway is compromised we can use *ex-utero* intrapartum treatment (EXIT procedure) to establish ventilation.^2^ Giancotti *et al.* reported a prenatally discovered mediastinal tumour with NIHF and resection one day after birth.^3^ In our case regular US were normal up to 30 weeks gestation. After spontaneous membrane rupture in 32 weeks gestation severe NIHF in geminus A and polyhydramnion were discovered. This indicates that tumour growth was fast with severe pressure on mediastinal structures.

Mediastinal teratomas without NIHF that were not discovered prenatally presented with severe respiratory distress in a live newborn after birth. In all cases surgery was performed with removal of the tumour, and mortality in this group was low.^9^–^13^

Postoperative course was complicated with severe tracheomalacia, wound sepsis and chyle leak in the Mogilner *et al*. case.^10^ Some authors described diaphragm paralysis with necessary plication after surgery.^3^,^6^ We found only one case in literature with prenatal collapse of the left bronchial system and tracheostomy after surgery at the age of 5 weeks.^6^

Seo *et al.* reported support with extracorporeal membrane oxygenation after surgery, but in this case a congenital cystic adenomatoid malformation, stocker type III (stocker-III CCAM) was later confirmed and the child had profound persistent fetal circulation postoperatively. Other authors do not mention using extracorporeal membrane oxygenation in children with mediastinal teratoma. In our case, the child had in addition to anatomical changes in the pulmonary vessels several risk factors for the development of chronic respiratory insufficiency including diaphragm paralysis, prematurity and several respiratory infections.

Heart catheterization in our case showed that tumour compression in prenatal period had an impact on the anatomy of mediastinal organs.

In conclusion, it is important to decide for early intervention if NIHF develops. Advances in radiologic imaging prenatally and after birth are the main tools for diagnostic evaluation of the disease. In cases when NIHF develops the outcome is mainly unpredictable because short and long term *sequelae* of combined effects of NIHF and teratoma pressure on neighboring organs may develop as we presented with our case.

## Figures and Tables

**TABLE 1.** Clinical neonatal successfull outcome of fetal mediastinal teratomas with hydrops fetalis

**Author/No.**	**Sex**	**Imaging**	**Prenatal procedure**	**Outcome**	**Histology**
Takayasu^4^	M	US (23 WG): Cystic formation in right anterior mediastinum MRI (29 WG): Cystic/solid mass, NIHF, polyhydramnion	Aspiration of the fetal tumour cyst fluid, Amniocentesis	Hydrops fetalis subsided No RD after birth Resection 30 days after birth NED	Mature teratoma
Giancotti^3^	M	US (29 WG): Anterior mediastinal mass MRI (31 WG): Anterior mediastinal mass, NIHF, polyhydramnion US (32 WG): Rapid growing mass	No	Elective cesarion section (32 WG) RD after birth, resection 1day after birth Left vocal cord/left diaphragm paralysis 18 day after birth diaphragm plication No respiratory problem	Teratoma
Merchant^2^	ND	US (21 WG): Anterior mediastinal mass MRI (22 WG): Anterior mediastinal mass with displacement of the heart, calcification, NIHF	*In utero* resection of the tumour	Preterm labour (25 WG) Bronchopulmonary dysplasia Well at home	Immature teratoma
Merchant^2^	ND	US/MRI (34 WG) Anterior mediastinal mass, calcifications, NIHF, polyhydramnion	Amnioreductions	EXIT procedure with tumour resection Well at home	Immature teratoma
Present case	M	US (33 WG) NIHF, polyhydramnion	No	Urgent cesarion section (33 WG) RD after birth, resection 7 days after birth Chronic respiratory insuficiency 8 month after procedure	Immature teratoma

M = male; MRI = magnetic resonance imaging; ND = no data; NED = no evidence of disease; NIHFb = non-immune hydrops fetalis; RD = respiratory distress; US = ultrasound; WG = weeks gestation
